# Supplementary material for: Surveillance of Viral Encephalitis in the Context of COVID-19: A One-Year Observational Study among Hospitalized Patients in Dakar, Senegal
Source: Viruses. 2022 Apr 22;14(5):871. doi: 10.3390/v14050871 (PMC9145710; doi:10.3390/v14050871)
Supplement: Supplementary file 1 [file viruses-14-00871-s001.zip › viruses-1631538-supplementary.pdf]

Supplementary Materials:

Table S1: List of viruses tested in this study, with the method and the reference.

Pathogen	Specimen type[1]	Method	*Reference and Sensitivity/LoD*[2]
Human Simplex Virus Type 1 (HSV-1)<br>Human Simplex Virus Type 2 (HSV-2)<br>Human Herpes Virus type 6 (HHV-6)<br>Human Herpes Virus type 7 (HHV-7)<br>Cytomegalovirus (CMV)<br>Epstein-Barr virus (EBV)<br>Varicella-Zoster Virus (VZV)	CSF	Allplex™ Meningitis-V1 assay IVD/CE (Real-Time PCR)	*Seegene Inc., Seoul, Republic of Korea (Sensitivity=100%)*
Human Parechovirus (HPeV)<br>Adenovirus (AdV)<br>Parvovirus B19 (B19V)<br>Mumps Virus (MV)<br>Human Enterovirus (HEV)	CSF	Allplex™ Meningitis-V2 assay IVD/CE (Real-Time PCR)	*Seegene Inc., Seoul, Republic of Korea (Sensitivity=96.88%)*
Dengue Virus (DENV)	Blood, CSF	Real-Time PCR	*[25] (Sensitivity=98.5%)*
Yellow Fever Virus (YFV)		Real-Time PCR	*[26] (Sensitivity=97.7%)*
Zika Virus (ZIKV)		Real-Time PCR	*[27] (Sensitivity=97.4%)*
West-Nile Virus (WNV)		Real-Time PCR	*[28] (LoD=10 copies/reaction)*
Rift Valley Fever Virus (RVFV)		Real-Time PCR	*[29] (Sensitivity=92.1%)*
Crimean-Congo Hemorrhagic Fever Virus (CCHFV)		Real-Time PCR	*[30] (not assessed)*
Chikungunya Virus (CHIKV)		Real-Time PCR	*[31] (LoD=27copies/reaction)*
SARS-CoV-2	NP, CSF	Real-Time PCR	*TIB Molbiol Syntheselabor GmbH, Berlin, Germany (Sensitivity=96.5%)*
Respiratory viruses panel: Influenza A (Flu A), Influenza B (Flu B), human rhinovirus (HRV), Respiratory Syncytial Virus (RSV), Metapneumovirus (MPV), Parainfluenza virus (PIV) and Adenovirus (AdV),	NP	FTD Respiratory pathogens 21 Assay (Real-Time PCR)<br>OR<br>Allplex™ RV Essential assay (Real-Time PCR)	*Siemens Healthcare GmbH, Erlangen, Germany (Sensitivity=93.94%)*<br><br>*Seegene, Seoul, South Korea (LoD=100-500 copies/reaction)*

[1]CSF = Cerebospinal fluid; NP = Nasopharyngeal

[2]Diagnostic sensitivity or limit of detection (LoD) are based on data available from published references or according suppliers.

Table S2: Neurological signs, extra-neurological signs, underlying condition and neurological assessment score at discharge of viral positive infected patients.

Clinical signs (N=27)	n (%)		n (%)
**Neurological signs**		**Extra-neurological signs**	
Impaired consciousness	14 (52)	Respiratory disorders	5 (19)
Motor deficit	10 (37)	Arthralgia/myalgia	5 (19)
Tetraplegia	4 (15)	Anosmia	3 (11)
Paraplegia	3 (11)	Delirium	1 (4)
Hemiplegia	3 (11)	hyperkeratotic skin lesions	1 (4)
Meningeal syndrome	10 (37)	Erythroderma	1 (4)
Behavioral change	8 (30)	**Underlying condition**	
Cranial nerve damage	8 (30)	HTA	5 (19)
III, IV, VI	6 (22)	HIV	3 (11)
VII	2 (7)	Unknown	19 (70)
Seizures	7 (26)	**LOS at discharge**[1]	
Intracranial hypertension	6 (22)	5	4 (15)
Spinal syndrome	3 (11)	4	6 (22)
Retrograde amnesia	2 (7)	3	2 (7)
Ataxia	2 (7)	2	4 (15)
Choreic abnormal movements	1 (4)	1 (death)	11 (41)

[1] LOS = Liverpool Outcome Score [32]. Outcome score: 5: full recovery; 4: minor sequelae with no effect, or only minor effects on physical function, or personality change, or on medication; 3: moderate sequelae mildly affecting function, probably compatible with independent living; 2: severe sequelae, impairing function sufficient to make patient dependent; 1: death.
